# Diagnosis and treatment of ectopic thyroid carcinoma: A case report and literature review

**DOI:** 10.3389/fonc.2022.1072607

**Published:** 2022-11-18

**Authors:** Guiming Fu, Fengli Guo, Wei Zhang, Xianhui Ruan, Xiangqian Zheng, Zhaohui Wang, Ming Gao

**Affiliations:** ^1^ Department of Thyroid and Neck Tumor, Key Laboratory of Cancer Prevention and Therapy, Tianjin’s Clinical Research Center for Cancer, Tianjin Medical University Cancer Institute and Hospital, National Clinical Research Center for Cancer, Tianjin, China; ^2^ Thyroid-otolaryngology Department, Sichuan Cancer Hospital & Institute, Sichuan Cancer Center · School of Medicine, University of Electronic Science and Technology of China, Chengdu, China; ^3^ Department of Thyroid and Breast Surgery, Binzhou Medical University Hospital, Binzhou, Shandong, China; ^4^ School of Medicine, Nankai University, Tianjin, China; ^5^ Department of Thyroid and Breast Surgery, Tianjin Union Medical Center, Tianjin, China; ^6^ Tianjin Key Laboratory of General Surgery in Construction, Tianjin Union Medical Center, Tianjin, China

**Keywords:** ectopic thyroid, thyroid tumor, thyroid cancer, diagnosis, treatment

## Abstract

**Introduction:**

Ectopic thyroid cancer (ETC) is primary thyroid cancer occurring in ectopic thyroid tissue, and its incidence rate is approximately 0.3%–0.5% of thyroid cancer. Only approximately 132 cases of ETC have been diagnosed and treated worldwide in the past 110 years, with most of them being adults. Of note, patients with ETC are prone to misdiagnosis and mistreatment.

**Case report:**

This was a 13-year-old adolescent female who reported having a sensation of swallowing obstruction when eating blocky foods. Color Doppler Ultrasound (CDU) found a 2.3 cm ×1.7 cm × 2.1 cm hypoechoic nodule slightly to the right of the deep surface of the tongue base, with a honeycomb shape. Meanwhile, a mixed echogenic nodule of approximately 2.0 cm × 1.9 cm × 2.3 cm was seen deep in the mouth floor, and a very low echogenic region of 1.4 cm × 1.1 cm × 1.8 cm was observed in the nodule. We then performed a fine needle aspiration biopsy (FNAB) of the thyroid nodules guided by CDU, and the results showed papillary thyroid carcinoma (PTC). Then, a local extended resection of the thyroid carcinoma was performed. Bilateral cervical IA and adjacent subhyoid lymph node dissection was performed through a small anterior cervical incision. The patient recovered well, and was discharged on the fifth day after surgery. The patient only took levothyroxine tablets for replacement therapy after surgery. The patient was followed up for 36 months, and the thyroid function remained in the normal range. Reexamination by CDU showed no tumor recurrence, lymph node enlargement, or obvious change in the tongue base ectopic thyroid.

**Conclusions:**

ETC is an extremely rare type of thyroid cancer, which is easy to be misdiagnosed. Preoperative use of CDU, nuclide scanning, computed tomography (CT)/Magnetic resonance imaging (MRI), and FNAB can significantly reduce the misdiagnosis rate of this disease. Surgery is currently the main treatment for ETC. Complete resection still has a high cure rate. For patients with advanced ETC who cannot be completely resected, external radiotherapy and targeted therapy can be tried, but the prognosis needs to be verified with more cases in the future.

## Introduction

The ETC refers to primary cancer occurring in ectopic thyroid tissue, and its incidence rate is approximately 0.3–0.5% of thyroid cancer (1). To our knowledge, only approximately 130 cases of ETC have been clearly diagnosed and treated worldwide in the past 110 years, with most of the cases occurring in adults. It is noteworthy that patients with ETC are prone to misdiagnosis and mistreatment. Herein, we report the diagnosis and treatment of the youngest adolescent with ETC to date. In addition, we summarized this ETC’s clinical manifestations, diagnosis, and treatment by reviewing previous relevant literature.

## Case report

The patient was a 13-year-old adolescent female who reported having a sensation of dysphagia when eating solid foods. The symptoms had been present for approximately 2 years, and the patient had no sore throat, hoarseness, dyspnea, or hemoptysis. By chance, the patient found a mass at the base of her tongue after inserting her fingers into her mouth. She had no past medical history. On physical examination, in addition to the raised mass at the base of the tongue, we found a mass just above the hyoid bone, approximately 2 cm in diameter, with poorly defined boundaries and poor mobility. No definite thyroid was found by palpation in the normal thyroid anatomy area, and no abnormal enlarged lymph nodes were found in the anterior neck and bilateral neck. Initially, the mass at the base of the tongue was the differentially diagnosed as lingual ectopic thyroid, hemangioma, or other possibilities, while the mass around the hyoid bone was diagnosed as a thyroglossal duct cyst, and surgery was recommended.

**Figure 1:**
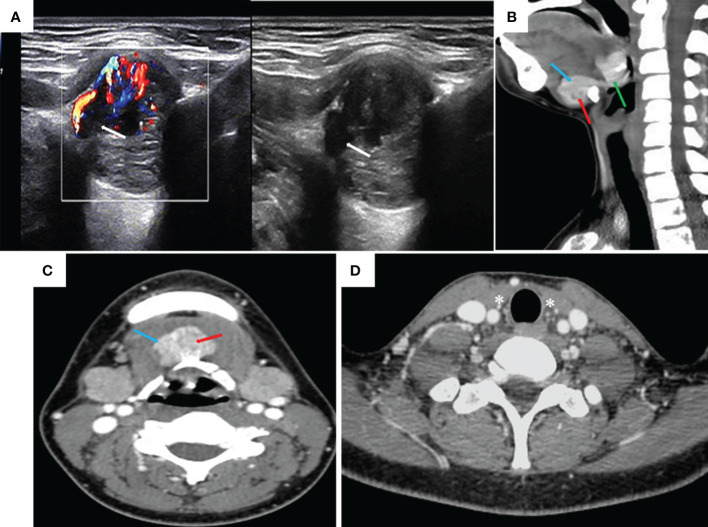
Color Doppler ultrasound and contrast-enhanced CT. **(A)** Ectopic thyroid and carcinoma nodule at the floor of the mouth (very hypoechoic area shown by the white arrow). **(B, C)** Ectopic thyroid gland at the base of the tongue, ectopic thyroid gland at the base of the mouth, and a single cancer nodule (the green arrow is the thyroid gland at the base of the tongue, the blue arrow is the thyroid gland at the base of the mouth, and the red arrow is a cancer nodule). **(D)** No thyroid gland in the normal position of the neck (white * area).

The patient underwent a series of tests after hospitalization. CDU found a hypoechoic nodule of 2.3 cm × 1.7 cm × 2.1 cm in size, slightly to the right of the deep surface of the tongue base, with a honeycomb shape (**Figure 1**). As a result, an ectopic thyroid was considered. Moreover, a mixed echogenic nodule of approximately 2.0 cm × 1.9 cm × 2.3 cm in size was found on the deep surface of the mouth floor. Its boundary was unclear, shape was irregular, it was connected to the tongue base mass, and its boundary with the surrounding muscle tissue was unclear (**Figure 1**). A very low echogenic region of 1.4 cm × 1.1 cm × 1.8 cm was observed in this nodule, suggesting the possibility of an ectopic thyroid with an ETC nodule. There were no abnormal swollen lymph nodes in the bilateral neck. No thyroid tissue was detected in the normal thyroid region. The findings of contrast-enhanced CT scan of the neck were similar to those of CDU (**Figure 1**). Thyroid function: free triiodinated thyroxine 3.13 pg/ml, free thyroxine 0.83 ng/dl, thyroid-stimulating hormone (TSH) 5.76 mIU/L, thyroglobulin 37.3 ng/ml, anti-thyroglobulin antibody < 5.00 IU/ml, anti-thyroid peroxidase antibody 2.37 IU/ml, parathyroid hormone 70.40 pg/ml, serum calcium < 0.50 pg/ml. Chest X-ray, electrocardiogram, and other auxiliary examinations found no specific abnormalities. Furthermore, we performed FNAB of thyroid nodules guided by CDU, and the results showed papillary thyroid carcinoma (PTC).

**Figure 2:**
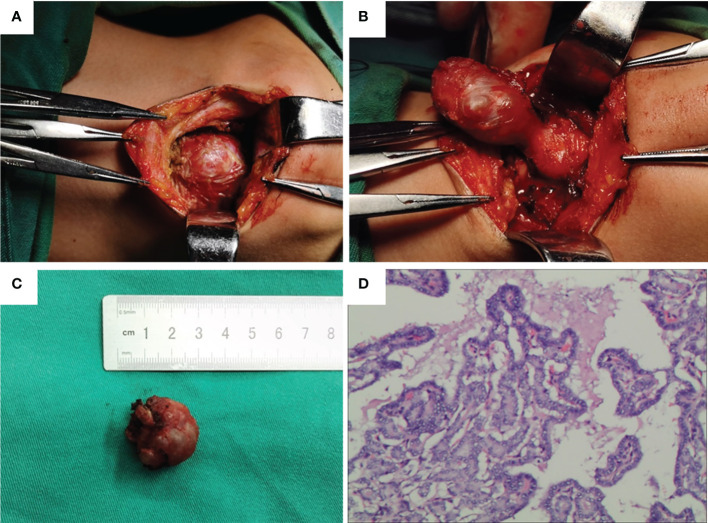
Intraoperative findings and postoperative pathology. **(A–C)** Ectopic thyroid and cancer foci in the floor of the mouth. **(D)** Postoperative pathology showed papillary thyroid cancer.

Subsequently, we performed a local extended resection of the thyroid carcinoma but preserved the normal thyroid tissue at the base of the tongue. Bilateral cervical IA and adjacent subhyoid lymph node dissection were performed through a small anterior cervical incision. During surgery, we found that the ETC nodule was wrapped by ectopic thyroid tissue on the floor of the mouth and connected with ectopic thyroid tissue at the base of the tongue (**Figure 2**). No parathyroid glands were found during the surgery. Postoperative pathology showed that the nodules in the ectopic thyroid tissue at the mouth floor were PTC (**Figure 2**), and 1 of 12 lymph nodes had metastasis. Parathyroid hormone measurements were performed on postoperative days 1 and 3, and the results were normal. The patient recovered well without neck bleeding, facial numbness, or convulsions, and was discharged on the fifth day after surgery. Levothyroxine tablet replacement therapy was started 7 days after surgery. Thyrotropin suppression and radioiodine-131 therapy were not performed.

To date, the patient has been followed up for 36 months, and the thyroid function and TSH are in the normal range. No tumor recurrence or suspicious lymph node metastasis was found by CDU of the neck, and there was no significant change in ectopic thyroid tissue at the base of the tongue. The patient reported being very satisfied with the therapeutic effect.

## Discussion

### Occurrence and classification of the ectopic thyroid gland

Ectopic thyroid gland is a rare congenital malformation. The incidence of ectopic thyroid in the general population is approximately 0.3/100,000-1/100000, and the incidence of thyroid disease is approximately 0.01%–0.03%, of which women account for approximately 65%–80%, significantly more than men (2, 3). The disease begins at the embryo stage of human development. In the fourth week of embryonic development, the endodermal epithelial cells between the ventral ends of the first and second pairs of branchial arches proliferate and sag to form a thyroid diverticulum, which further extends into the thyroglossal canal. The thyroglossal canal may extend through the body of the tongue in the anterior direction of hyoid bone and thyroid cartilage to gradually form a solid cell cord. Ectopic thyroid occurs when the cell cord descends abnormally into the 2nd–4th ring of the trachea. Ectopic thyroid can be seen in almost any part of the body. Over 90% of them occur in the tongue (4, 5), followed by the sublingual area, under the jaw area (6), before and after the hyoid bone, the lateral neck, trachea and parotid gland, axillary region, or heart (7–11). This is consistent with our statistical results for ETC. According to the presence or absence of thyroid tissue in the normal anatomical position of the neck, the ectopic thyroid can be divided into two categories: vagal thyroid (absence of thyroid in the normal position and presence of thyroid in the abnormal position) and extra thyroid gland (presence of thyroid in both the normal and abnormal positions).

### Manifestations and diagnosis of ETC

The severity of symptoms in the vast majority of patients with ETC is related to the tumor size and anatomical location. When the tumor is large enough, events such as local compression or intracavitary obstruction will occur. When ETC occurs in the head and neck, patients may also present with dizziness, vomiting, sore skin, snoring, hoarseness, irritating cough, dyspnea, sore throat, swallowing obstruction, head and face swelling, and other symptoms (12–15), and it needs to be distinguished from thyroglossal duct cyst (15–17), hemangioma, dermoid cyst, lymphatic tuberculosis, papilloma of the nasal cavity (18), nasopharyngeal carcinoma (19), laryngeal tumor (20, 21), pharyngeal tumor (22), esophageal tumor, lymph node metastasis cancer, and other diseases (23). When ETC occurs in the chest and mediastinum, it can be accompanied by hoarseness, dyspnea, eating disorders, and chest deformity, and it should be differentiated from thymoma, teratoma, tracheal tumor, esophageal cancer, and even bone tumor of the chest wall (24–29).. When ETC occurs in the abdominal cavity or pelvic cavity, symptoms such as abdominal pain, abdominal distention, loss of appetite, anemia, defecation disorders (30), and even recurrent urinary tract infections may occur, and it needs to be differentiated from primary and secondary tumors originating from the abdomen and pelvic cavity (31–34).

Among the previously reported ETC cases, the detection rates of CDU, CT/MRI, Radioiodine scan, and FNAB were 81.1%, 78.8%, 20.5%, and 41.0%, respectively. Approximately 60% of the patients were not clearly diagnosed before surgery (**Table 1**). For patients with suspected ETC, it is important to select appropriate auxiliary examinations in addition to physical examinations. The proportion of patients with ectopic thyroid (or thyroid cancer) complicated with hypothyroidism is relatively high, up to approximately 30%, as reported in the literature (35). Routine assessment of thyroid hormone levels can determine whether patients have hyperthyroidism or hypothyroidism in advance, which can effectively avoid the occurrence of postoperative hyperthyroidism crisis and reduce the risk of hospitalization. We believe that, as with thyroid cancer surgery in general, preoperative and postoperative parathyroid detection may help to better compare the changes in parathyroid function. If necessary, doctors can even choose to perform a 99mTc-sestamibi single-photon emission computed tomography (99mTc-MIBI SPECT) scan before surgery to determine the location and number of parathyroid glands, thereby avoiding the occurrence of permanent parathyroid dysfunction. To the best of our knowledge, although PTC co-exists in over 90% of ETC, medullary thyroid carcinoma has also been reported (36). Thus, calcitonin detection is also necessary. As the preferred method of ETC diagnosis, CDU can confirm the presence of thyroid in the normal position and also make a preliminary judgment on the size, shape, boundary, blood flow, and anatomical relationship with the surrounding structures of the suspicious mass. It should be noted that CDU cannot independently diagnose the presence of ETC but needs to be combined with thyroid radionuclide scanning and FNAB. In particular, CDU is more limited in the diagnosis of ETC in the mediastinum, abdominal cavity, and other parts. When the mass is large, the CDU diagnosis is unclear, or CDU considers ETC as a suspicious mass. CT/MRI scan can clearly show the size, shape, boundary, and the relationship between the surrounding structure of the mass from multiple levels as well as roughly assess the characteristics of the mass according to its tissue density and signal strength (26, 27, 29). When the tumor is located in the mediastinum, chest, or abdominal cavity, CT/MRI can also be selected to determine whether there are adhesions or any infiltration with the surrounding great vessels, to fully evaluate the surgical risk (37). In case CDU fails to identify the presence of the thyroid, iodine-131 or ^99^Tcm radionuclide scanning can be very effective for identifying normal or ectopic thyroid tissue and can also clearly display metastatic lesions. However, it is worth noting that some ectopic thyroid functions are low or nonfunctional, and the radioactive nuclide uptake is not obvious, which may lead to misdiagnosis. However, this test is not recommended for routine use because of radiation exposure. To avoid misdiagnosis of the disease, preoperative FNAB guided by CDU can be selected (38), and intraoperative freezing is also feasible. Besides, thyroid globulin levels can be measured to assist in diagnosis when medical conditions allow. When ETC is located in the nasopharynx, trachea, esophagus, cervix, and other special sites, endoscopic assistance can often achieve the purpose of biopsy. For patients with ETC and distant metastasis, whole body bone scans and PET-CT can determine the location and number of metastases. In 2017, Hu et al. reported the first case of ectopic mediastinal PTC using endobronchial ultrasound-guided transbronchial needle aspiration (EBUS-TBNA) for safe and accurate sampling, which we believe is worthy of recommendation (26).

**Table 1:** The diagnose and treatment of 132 patients with ectopic thyroid carcinoma.

Area		Number	Diagnose						Treatment				Relapse or metastasis	Total
			CDU	CT/MRI	RAI-S	FNAB	Others		Surgery	RAI-T	RAD	Others		
Head and maxillofacial														6
	Scalp	1	–	1	–	–	–		1	–	–	–	–	
	Skull	2	–	2	–	–	–		2	–	–	–	1	
	Nasopharynx, nasal Septum	2	–	2	–	–	2		2	–	–	–	–	
	Parotid gland	1	–	–	–	–	–		1	–	–	–	1	
Oral cavity, pharynx and neck														107
	Tongue, pharyngeal	63	56	52	18	27	18		59	13	6	5	5	
	Midline of the neck (trachea, larynx, esophagus, etc)	32	27	22	7	15	9		32	7	1	3	2	
	Lateral neck	12	10	8	–	3	–		12	6	–	–	1	
Chest														11
	Mediastinum (esophagus, pericardium, etc.)	8	6	8	1	4	5		8	2	2	2	1	
	Clavicle and chest wall	3	1	2	1	2	2		3	1	2	1	1	
Abdominal and pelvic														8
	Abdominal cavity (liver, rectum)	2	1	2	–	2	2		2	1	–	1	2	
	Pelvic cavity (uterus, ovaries, etc)	6	6	5	–	1	2		6	2	–	1	2	
Total		132	107	104	27	54	40		128	32	11	13	16	132

CDU, Color Doppler ultrasound; CT/MRI, Computed tomography/Magnetic resonance imaging; RAI-S, Radioiodine scan; FNAB, Fine needle aspiration biopsy; RAI-T, Radioiodine-therapy; RAD, Radiotherapy.

### The treatment of ETC

Herein, we summarized the previous treatment methods of 132 ETC patients (**Table 1**) by reviewing the literature. The proportion of ETC patients receiving surgery, radioiodine, and external radiotherapy was 97.0%, 24.2%, and 8.3%, respectively. The rate of tumor recurrence and metastasis was 12.1%, and only a few studies reported a survival time. We noted that surgery and radioiodine are still the main treatment methods for ETC. A small number of patients with advanced ETC received external radiotherapy or targeted therapy, but the prognosis was poor, and the benefit was little (33). For the surgical treatment of ETC, preoperative consideration should be made based on the classification of the ectopic thyroid, pathological types of cancer foci (e.g., follicular carcinoma, medullary carcinoma, poorly differentiated or undifferentiated carcinoma), location and size of the cancer foci, patient age, and other factors. For patients requiring iodine-131 postoperative treatment, not only the ectopic thyroid gland should be completely removed, but the normal anatomical location should also be checked for the presence of glands, and if any, the gland should also be removed (39, 40). It should be noted that for ETC located at the base of the tongue and the floor of the mouth, postoperative wound bleeding could easily lead to airway obstruction, and a preoperative temporary tracheotomy should be considered. In the existing case reports, only a few scholars have carefully described the extent of intraoperative lymph node dissection and the number of lymph node metastases. It is still highly controversial whether prophylactic neck dissection or only therapeutic lymph node dissection should be performed in patients with ETC. We found that some scholars did not perform cervical lymph node dissection when removing the primary tumor lesions, and the patients still achieved long-term disease-free survival (41, 42). The extent of lymph node dissection should be determined according to the specific body location and the pathological type of the tumor. For lesions with a low risk of metastasis and recurrence, lymph node dissection may increase the incidence of complications. When dealing with adolescent patients, the formulation of a surgical plan needs to be more cautious, and endocrine dysfunction in the process of growth and development should be avoided as much as possible while taking into account the radical treatment of cancer. The adolescent patient with ectopic thyroid cancer reported in this paper had a small tumor and no obvious extracapsular extension, which is considered to be at low risk of recurrence. Neck lymph node dissection was unnecessary, but the patient’s mother strongly requested neck lymph node dissection before surgery. After consultation, we only performed bilateral neck dissection of the IA and subhyoid regions through the small anterior cervical incision, and the final pathology showed that only one metastatic lymph node existed. Finally, attention should be paid to identifying and protecting the parathyroid gland during ETC resection to avoid the occurrence of permanent hypoparathyroidism. Postoperative radioiodine therapy and TSH suppression can be referred to as the treatment of thyroid cancer in general.

## Conclusion

ETC is an extremely rare type of thyroid cancer, which is easily misdiagnosed. Preoperative use of CDU, nuclide scanning, CT/MRI, and FNAB can significantly reduce the misdiagnosis rate of this disease. Surgery is currently the main treatment for ETC, and complete resection still has a high cure rate. For patients with advanced ETC who cannot be completely resected, external radiotherapy and targeted therapy can be attempted, but the prognosis needs to be verified with a larger sample in the future.

## Data availability statement

The original contributions presented in the study are included in the article/supplementary material. Further inquiries can be directed to the corresponding authors.

## Ethics statement

Written informed consent was obtained from the individual(s), and minor(s)’ legal guardian/next of kin, for the publication of any potentially identifiable images or data included in this article.

## Author contributions

GF and FG were responsible for writing the manuscript. GF was responsible for the collection and sorting of patient data. WZ searched and collected a large number of relevant literature. XR and XZ gave professional advice on the writing. ZW and MG made important revisions to the manuscript, gave the final approval to publish the manuscript, and agreed to be responsible for all aspects of the work to ensure that issues relating to the accuracy or completeness of any part of the work are properly investigated and resolved. All authors contributed to the article and approved the submitted version.

## Funding

This work was supported by grants from the National Natural Science Foundation of China (81872169,82172821,82103386), Tianjin Municipal Science and Technology Project (19JCYBJC27400,21JCZDJC00360) and Beijing-Tianjin-Hebei Basic Research Cooperation Project(20JCZXJC00120), The Science &Technology Development Fund of Tianjin Education Commission for Higher Education(2021ZD033),Tianjin Medical Key Discipline(Specialty) Construction Project(TJYXZDXK-058B),Tianjin Health Research Project(TJWJ2022XK024).

## Acknowledgments

Thanks to the English editing Services from Charlesworth Author Services.

## Conflict of interest

The authors declare that the research was conducted in the absence of any commercial or financial relationships that could be construed as a potential conflict of interest.

## Publisher’s note

All claims expressed in this article are solely those of the authors and do not necessarily represent those of their affiliated organizations, or those of the publisher, the editors and the reviewers. Any product that may be evaluated in this article, or claim that may be made by its manufacturer, is not guaranteed or endorsed by the publisher.
